# Persisting thrombomodulin resistance at 3 months after liver transplantation in children with cirrhosis

**DOI:** 10.1016/j.rpth.2025.102709

**Published:** 2025-02-27

**Authors:** Marie-Astrid van Dievoet, Clara David, Audrey Dieu, Cedric Hermans, Thierry Pirotte, Jonathan Douxfils, Ton Lisman, Xavier Stephenne

**Affiliations:** 1 Laboratory of Pediatric Hepatology and Cell Therapy, Institut de Recherche Expérimentale et Clinique (IREC), Université catholique de Louvain, Brussels, Belgium; 2 Laboratory Department, Cliniques Universitaires Saint-Luc, Brussels, Belgium; 3 Clinical Pharmacology and Toxicology Research Unit, Namur Research Institute for Life Sciences (NARILIS), University of Namur, Namur, Belgium; 4 Research and Development Department, QUALIblood s.a., QUALIresearch, Liège, Belgium; 5 Department of Anesthesiology, Cliniques Universitaires Saint-Luc, 1200 Brussels, Belgium; 6 Haemostasis and Thrombosis Unit, Division of Haematology, Cliniques Universitaires Saint-Luc, Brussels, Belgium; 7 Surgical Research Laboratory and Section of Hepatobiliary Surgery and Liver Transplantation, University of Groningen, University Medical Center Groningen, Groningen, Netherlands; 8 Division of Pediatric Gastroenterology and Hepatology, Department of Pediatrics, Cliniques Universitaires Saint-Luc, Rare Liver ERN, Transplantchild ERN, Brussels, Belgium

**Keywords:** liver cirrhosis, liver transplantation, pediatrics, thrombomodulin, thrombin generation

## Abstract

**Background:**

The coagulation cascade in pediatric cirrhotic patients appears rebalanced, similar to adults, with few true hemostasis-related bleeds or thromboembolic events before liver transplantation. Vascular thrombosis is an important post–liver transplantation complication. Few papers have addressed the recovery of the coagulation cascade after liver transplantation.

**Objectives:**

We aimed to assess the coagulation cascade, with both measurement of individual factors and a global hemostasis assay, before living donor liver transplantation and to investigate its recovery 3 months after transplantation, when liver function has normalized.

**Methods:**

From January 2022 to July 2023, pediatric cirrhotic patients were prospectively enrolled 1 day before liver transplantation. An age-matched control group was included for comparison. Routine hemostasis tests, levels of coagulation factors and natural anticoagulants, and thrombomodulin-modified thrombin generation were determined on automated coagulation analyzers at inclusion and 3 months after liver transplantation.

**Results:**

Twenty-seven pediatric patients with cirrhosis, primarily of cholestatic origin, and 10 controls were enrolled. Sixteen patients were sampled 3 months after liver transplantation. Pediatric end-stage liver disease scores ranged from −10 to 44. A rebalanced coagulation cascade was confirmed in cirrhotic children, indicated by a thrombomodulin-modified thrombin generation assay similar to controls, although with higher interpatient variability. Interestingly, 3 months posttransplant, coagulation was not completely normalized. In the majority of patients resistance to thrombomodulin persisted.

**Conclusion:**

This study confirmed a rebalanced coagulation system in pediatric cirrhotic patients before liver transplantation. Three months posttransplant thrombomodulin resistance persisted. Whereas this contributes to thrombotic complications observed after liver transplantation, remains to be elucidated.

## Essentials

• Limited literature on coagulation recovery after liver transplant (LT) in pediatric cirrhosis.
• Evaluation of coagulation factors and a global hemostasis assay before and after LT.
• Rebalanced coagulation confirmed, but with high variability among patients.
• Persisting thrombomodulin resistance 3 months after LT.

## 1 Introduction

The concept of rebalanced hemostasis is well-established in the adult cirrhotic population. Although the literature is more limited, it appears that the hemostatic system in pediatric patients with cirrhosis is also rebalanced in a similar manner [1,2]. This is in line with the paucity of true hemostasis-related bleeds in these patients. Most bleeding events pretransplant are variceal in nature and are mainly caused by high portal pressure. Similarly, thrombotic events are rarely seen in this patient group before liver transplantation.

Vascular thrombosis and bleeding are, however, important posttransplantation complications. The incidence of hepatic artery thrombosis after liver transplantation varies between 3.6% and 7.4% and presents a major cause of graft loss [3, 4, 5]. The actual cause of hepatic artery thrombosis is mostly unknown, but surgical risk factors, like technical issues with the arterial anastomosis, are mainly identified. In our center, a 1-year incidence of 5.7% was observed for portal vein thrombosis [5]. Portal vein thrombosis is associated with several risk factors: patient’s age, weight, biliary atresia, warm ischemia time, and technical variant grafts [6]. Peripheral thromboembolism is well-documented in adult patients but is less commonly reported in pediatric patients [7]. Deep venous thrombosis was seen in 6.5% of pediatric patients (*n* = 92) after liver or multivisceral transplantation in a study by Borst et al. [8]. Although surgical factors are recognized as risk factors for vascular complications, nonsurgical factors, like hypercoagulability, may also play a role [9,10]. Furthermore, a decrease in the rate of venous thrombosis was seen after the introduction of early postoperative heparin in a before and after study design [11]. The majority of bleeding events post–liver transplantation are surgical and often require a repeat intervention.

Few papers have addressed the recovery of the coagulation cascade following liver transplantation in children with cirrhosis. In those papers [1,12,13], coagulation parameters were assessed 4 to 6 weeks after liver transplantation. In our study, we aimed to assess the coagulation cascade, with both measurement of individual factors and a global hemostasis assay, before liver transplantation and to investigate its recovery 3 months afterward.

## 2 Methods

### 2.1 Patient population

From January 2022 to July 2023, pediatric patients with cirrhosis were prospectively enrolled 1 day before living donor liver transplantation at Cliniques Universitaires Saint-Luc (Brussels, Belgium). Informed consent was obtained from the parents or legal guardians. Blood samples were collected from the patients both immediately before and 3 months after liver transplantation. An age-matched control group of healthy individuals undergoing minor surgery was also included. The study received approval from the local ethics committee (2020/12MAR/157) in accordance with the Declaration of Helsinki.

### 2.2 Blood draw

Blood was collected in a citrate tube (Monovette plastic 3.0 mL, 3.2% citrate, 106 mmol/L, Sarstedt) after drawing a discard tube. Samples were homogenized immediately after sampling. Platelet-poor plasma was prepared within 30 minutes of venipuncture through 2 centrifugation steps (2 × 15 minutes at 2,500 g). After the first centrifugation, the plasma was decanted and subjected to a second centrifugation. The aliquots were then immediately stored at −80 °C until analysis.

### 2.3 Analysis

Studied parameters were measured using an automated coagulation analyzer, the ACL-TOP 750 (Werfen). Routine parameters were measured with the following reagents: RecombiplastIN 2G (Werfen) for prothrombin time, SynthASil (Werfen) for activated partial thromboplastin time, HemosIL Thrombin time for thrombin time and QFA Thrombin (Werfen) for fibrinogen. For the determination of the natural anticoagulants, the following assays were executed: chromogenic protein C (HemosIL Protein C), free protein S (HemosIL Free Protein S), and antithrombin (HemosIL Liquid Antithrombin). Intrinsic coagulation factors were measured using factor-deficient plasma (VIII, IX, and XI), and SynthASil (Werfen). Extrinsic coagulation factors were measured with factor-deficient plasma (II, V, VII, X) and RecombiplastIN 2G (Werfen). For all the parameters, calibration was performed with HemosIL calibration plasma, which is traceable to international standards.

The ST Genesia is a benchtop analyzer for thrombin generation measurements. Several differences with the calibrated automated thrombogram (CAT) method can be pointed out. This system is fully automated and allows continuous loading of reagents and samples. As with the CAT system, the ST Genesia system needs a calibration, but this can be done daily instead of being processed for each plasma on each plate. To allow this independent calibration, an additional evaluation of the absorption properties of the plasma is needed. The algorithm, developed initially for the CAT system to transform the fluorogenic signal into thrombin concentration, has been revised accordingly to integrate this new calibration principle.

Calibration was performed with STG-ThrombiCal, STG-FluoSet, and STG-FluoStart. Patient samples were run 2 times: once for the plasma absorption properties adjustment (STG-FluoSet) and once for the actual measurement of thrombin generation using STG-ThromboScreen and STG-FluoStart. The STG-ThromboScreen kit enables to run thrombin generation without and with thrombomodulin (TM) with a fixed concentration of tissue factor and phospholipids. Three levels of controls (STG-QualiTest) were also run.

### 2.4 Statistical analysis

Statistical analysis was performed with GraphPad Prism, version 9.5.1. Normal distribution was checked with a Kolmogorov–Smirnov test. For comparison between patients and control subjects, an unpaired Student’s *t*-test (normal distribution) or Mann–Whitney-U test (nonparametric distribution) was used. For comparison before and after liver transplantation, a paired Student’s *t*-test (normal distribution) or Wilcoxon test (nonparametric distribution) were performed.

## 3 Results and discussion

### 3.1 Patient description

Twenty-seven pediatric patients were enrolled prospectively, and a blood sample was taken 1 day (IQR: 1-7 days) before living donor liver transplantation. Sixteen patients were sampled 3 months afterward (IQR: 85-92 days). A control group, consisting of 10 age-matched, healthy pediatric controls, was also included. The majority of patients had cholestatic cirrhosis: biliary atresia (67%), progressive familial intrahepatic cholestasis (7%), and cholestatic cirrhosis of unknown etiology (11%). Other causes included α-1 antitrypsin deficiency (4%), drug-related cirrhosis (4%), and chronic liver disease of unknown etiology (7%). The median age was 21 months (IQR: 12-48) for patients and 20 months (IQR: 11-43) for controls. The pediatric end-stage liver disease (PELD) score varied between −10 and 44.

### 3.2 A rebalanced coagulation cascade is confirmed in children with liver cirrhosis

**Figure 1:**
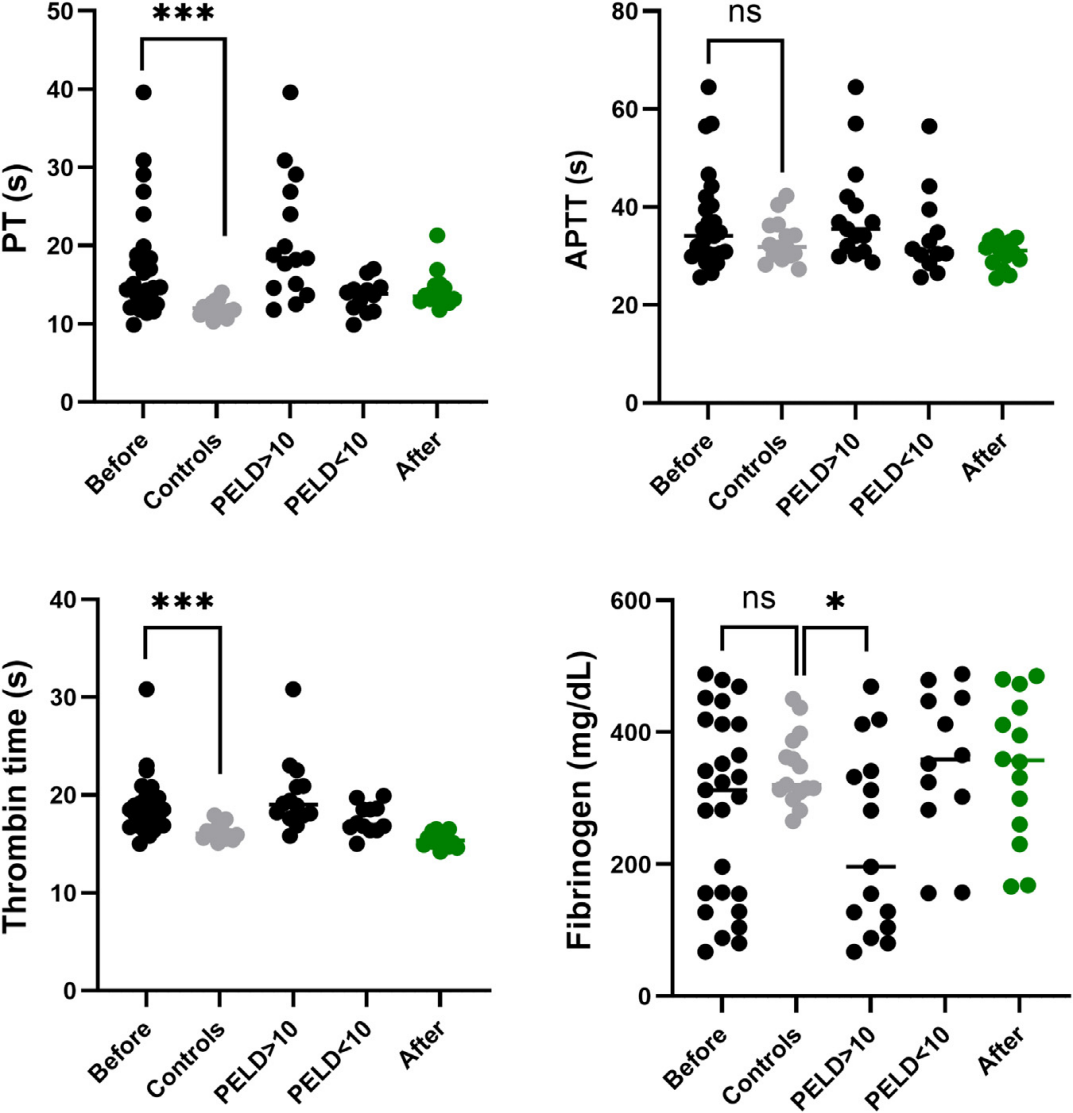
Routine hemostasis assays in patients before and after liver transplantation, compared with controls. Levels of significance: ns, not significant; ∗*P* < .05; ∗∗∗*P* < .001. Note the difference in prothrombin time (PT) and fibrinogen between patients with a pediatric end-stage liver disease score (PELD)<10 and >10. APTT, activated partial thromboplastin time. Bullets in black: patients before transplantation; bullets in green: patients after transplantation; and bullets in gray: age-matched control group.

**Figure 2:**
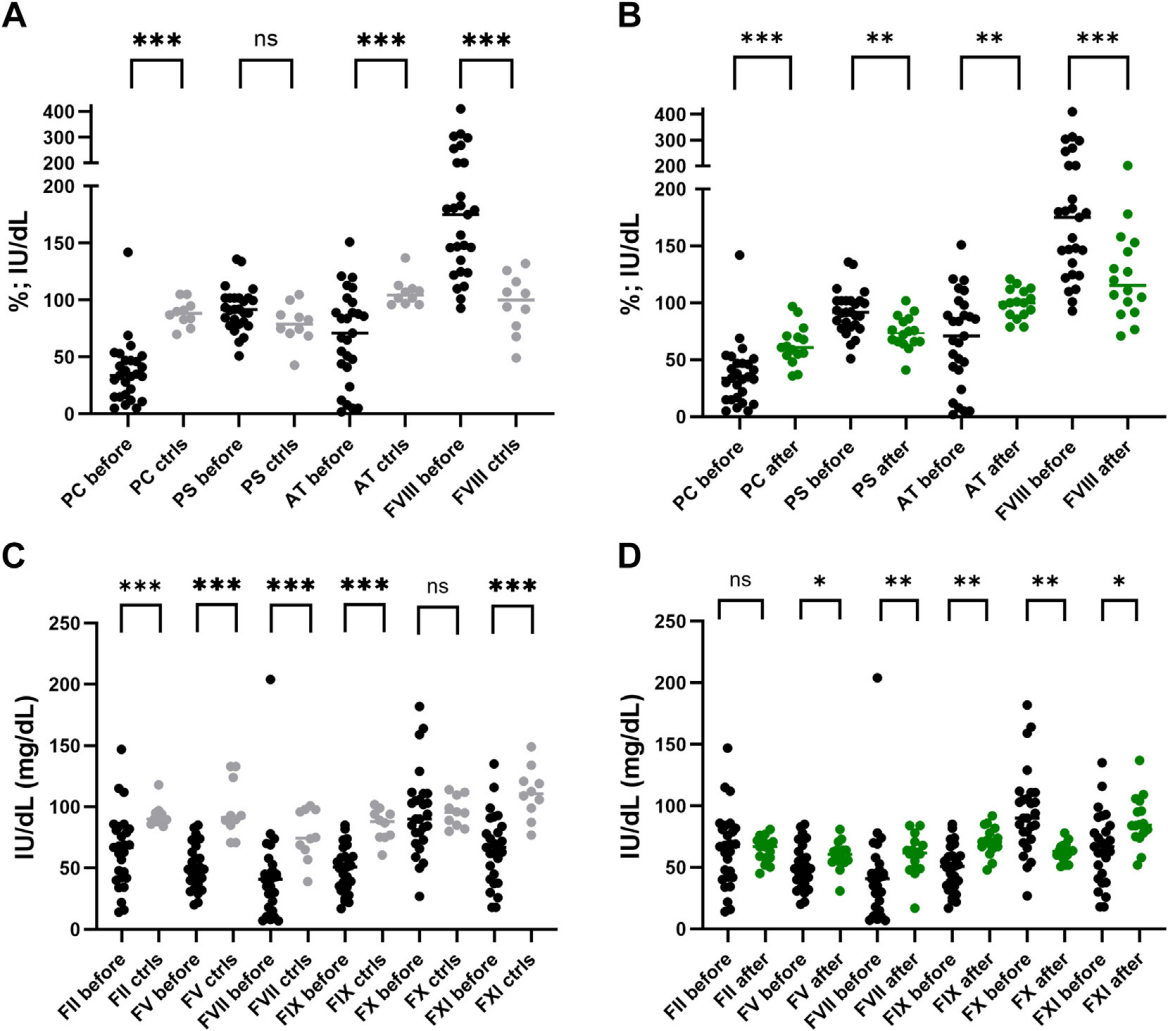
Anticoagulant drivers compared with controls (A) and after liver transplantation (B) and procoagulant drivers when compared with controls (C) and after liver transplantation (D). Levels of significance: ns, not significant; ∗*P* < .05; ∗∗*P* < .01; ∗∗∗*P* < .001. AT, antithrombin; ctrls, controls; F, factor; PS, protein S; PC, protein C. Bullets in black: patients before transplantation; bullets in green: patients after transplantation; and bullets in gray: age-matched control group.

Prothrombin time and thrombin time were significantly prolonged in patients compared with controls. In contrast, activated partial thromboplastin time and fibrinogen levels showed no significant differences, though there was greater interindividual variability among patients, particularly in fibrinogen. Perturbation of routine hemostasis assays was more pronounced in patients with higher PELD score (Figure 1). Despite these prolonged hemostasis assays, patients presented a rebalanced coagulation cascade with a simultaneous decline in procoagulant and anticoagulant proteins. This can be appreciated when looking at procoagulant and anticoagulant drivers of hemostasis (Figure 2). Except for factor VIII, coagulation factors were generally lower in patients when compared with controls. Natural anticoagulants, protein C and antithrombin, were significantly reduced, while protein S remained normal in most patients. Coagulation parameters showed considerable variability among patients compared with controls, consistent with findings from routine hemostasis assays.

**Figure 3:**
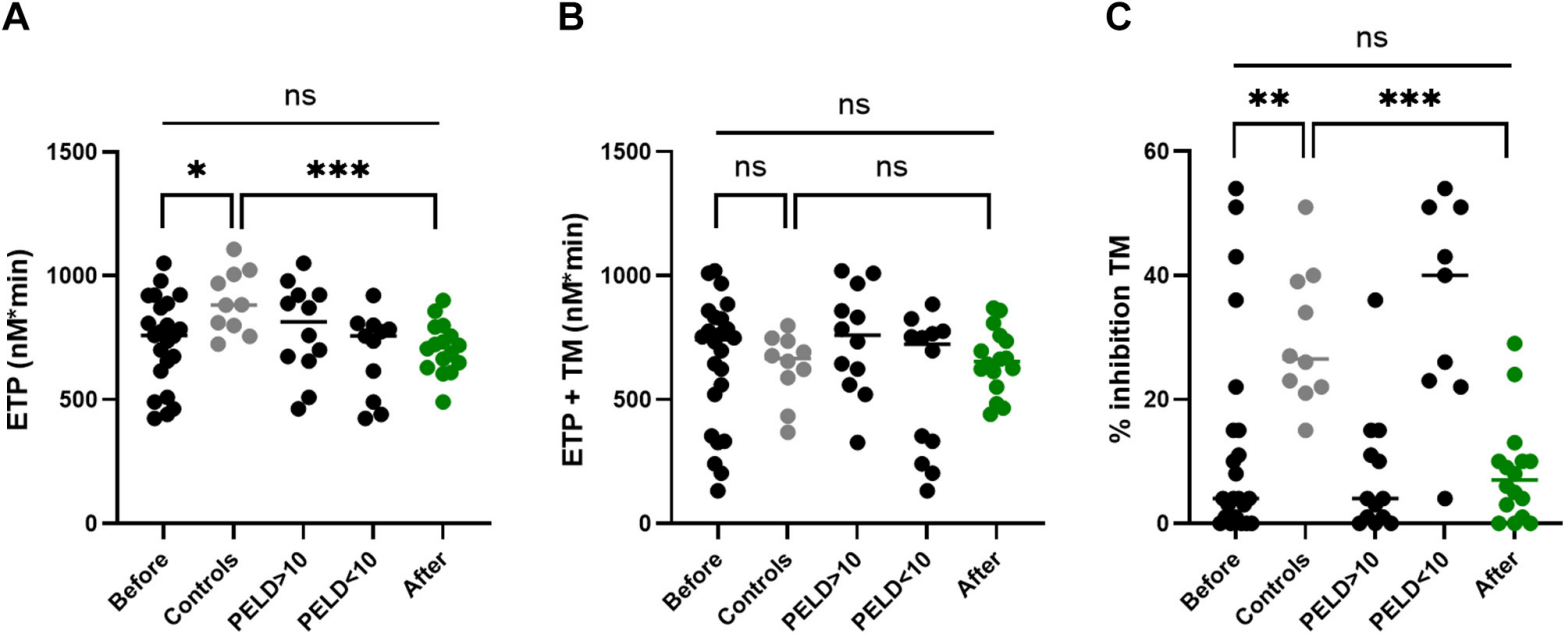
Thrombin generation (endogenous thrombin potential [ETP]) in patients compared with controls. (A) thrombin generation without thrombomodulin (TM); (B) thrombin generation with TM; and (C) percentage inhibition TM. Note: high resistance to TM in patients with high pediatric end-stage liver disease score (PELD) score and patients 3 months after liver transplantation. Levels of significance: ns, not significant; ∗*P* < .05; ∗∗*P* < .01; ∗∗∗*P* < .001. Bullets in black: patients before transplantation; bullets in green: patients after transplantation; and bullets in gray: age-matched control group.

Without TM, thrombin generation (endogenous thrombin potential) was lower in patients when compared with controls. In the presence of TM, no significant difference in endogenous thrombin potential was observed between the 2 groups, although variability between patients was high with some patients showing hypocoagulable or hypercoagulable features. When evaluating percentage inhibition TM, which measures the percentage inhibition of thrombin generation in the presence of TM, most patients with severe liver disease (indicated by a high PELD score) showed TM resistance. Protein C (r = 0.61) and protein S (r = 0.61) showed a good correlation with the percentage inhibition by TM, whereas FVIII did not demonstrate an inverse correlation with percentage inhibition (r = 0.27). Werner et al. [1] examined various hemostatic parameters before, during, and up to 1 month after liver transplantation in children with cirrhosis. As seen in our study, the endogenous thrombin potential was comparable to age-matched controls before liver transplantation. Magnusson et al. [14] found lower endogenous thrombin potential in children with prolonged routine coagulation assays, though they did not incorporate TM in their assay. Detailed results of thrombin generation are shown in Figure 3. Other thrombin generation parameters—lag time, time-to-peak, peak and velocity index—were not significantly different between patients and controls, although higher variability was shown between patients when compared with controls (Supplementary Figure 1).

### 3.3 Three months after liver transplantation, coagulation assays were not completely normalized

In 16 patients, coagulation cascade analysis was also performed 3 months after liver transplantation. Routine hemostasis parameters mostly normalized (Figure 1). Protein C and antithrombin were significantly higher 3 months after liver transplantation, but they remained lower than in the control group, especially protein C. The same trend was seen for the coagulation factors. The FVIII decreased after transplantation but remained higher when compared with controls. The remaining coagulation factors, with the exception of FII, were significantly higher in patients after liver transplantation but did not return to levels seen in the control group (Figure 2). Endogenous thrombin potential with TM improved posttransplantation with lower variability between patients. Interestingly, even 3 months after liver transplantation, patients still showed TM resistance (Figure 3). This may be partially attributed to persistently low levels of protein C (r = 0.31) and elevated FVIII (r = −0.13), although the correlation for protein C was not as strong as that observed before liver transplantation. This is in contrast to a paper by Mimuro et al. [13] where protein C activity was already normalized at 1 month post–liver transplantation. Similar to our study, their study cohort comprised a majority of biliary atresia patients undergoing living donor liver transplantation. Lisman et al. [12] sampled 11 pediatric patients, mostly biliary atresia patients, 37 days after transplantation and found slightly higher protein C levels (median of 64%; range: 51% to 105%), compared with our cohort posttransplant (median 61%; range: 36% to 97%). In addition to persistence of thrombomodulin resistance, shorter time-to-peak and higher velocity index were seen in patients after transplantation compared with controls (with and without thrombomodulin). Peak thrombin generation was slightly higher in patients after transplantation in the presence of thrombomodulin (Supplementary Figure 1).

In an adult cohort with cirrhosis, TM resistance was identified as an independent risk factor of portal venous thrombosis [15]. In this study, it is however, not clear if endogenous thrombin potential with TM was higher in patients compared with controls. In our study, virtually all patients who underwent sampling 3 months post–liver transplantation had resistance to TM in comparison to controls. It would be interesting in the future, larger studies to correlate the incidence of TM resistance to the incidence of vascular complications. In our study only 1 patient had hepatic artery thrombosis and died 1 month after liver transplantation. No portal vein thrombosis or peripheral thromboembolism was observed.

## 4 Conclusion

Rebalanced hemostasis was confirmed in a cohort of pediatric patients with cirrhosis, just before liver transplantation. Interestingly, 3 months after liver transplantation, coagulation was not completely normalized. In the majority of patients, resistance to TM persisted. Whereas this contributes to thrombotic complications observed after liver transplantation remains to be elucidated.
