# Supplementary material for: Persisting thrombomodulin resistance at 3 months after liver transplantation in children with cirrhosis
Source: Res Pract Thromb Haemost. 2025 Feb 27;9(2):102709. doi: 10.1016/j.rpth.2025.102709 (PMC12034219; doi:10.1016/j.rpth.2025.102709)
Supplement: Supplementary Figure 1 [file mmc1.docx]

**Fig. S1: Thrombin generation parameters without and with thrombomodulin in patients, before and after liver transplantation, compared to controls.** Levels of significance: ns, not significant; *p<0.05; **p<0.01; ***p<0.001. Abbreviations: PELD, pediatric end-stage liver disease score; TM, thrombomodulin; VI, velocity index. Bullets in black: patients before transplantation; bullets in green: patients after transplantation; bullets in grey: age-matched control group.
